# Behaviour of PMMA Resin Composites Incorporated with Nanoparticles or Fibre following Prolonged Water Storage

**DOI:** 10.3390/nano11123453

**Published:** 2021-12-20

**Authors:** Abdulaziz Alhotan, Julian Yates, Saleh Zidan, Julfikar Haider, Carlos Alberto Jurado, Nikolaos Silikas

**Affiliations:** 1 Division of Dentistry, School of Medical Sciences, University of Manchester, Manchester M13 9PL, UK; julian.yates@manchester.ac.uk (J.Y.); j.haider@mmu.ac.uk (J.H.); nikolaos.silikas@manchester.ac.uk (N.S.); 2 Dental Health Department, College of Applied Medical Sciences, King Saud University, Riyadh 11454, Saudi Arabia; 3 Department of Dental Materials, Faculty of Dentistry, Sebha University, Sebha 18758, Libya; sal.zidan@sebhau.edu.ly; 4 Department of Engineering, Manchester Metropolitan University, Manchester M1 5GD, UK; 5 Woody L. Hunt School of Dental Medicine, Texas Tech University Health Sciences Centre El Paso, 5001 El Paso Drive, El Paso, TX 79905, USA; carlos.jurado@ttuhsc.edu

**Keywords:** PMMA denture base, zirconia (ZrO_2_), titania (TiO_2_), e-glass fibre, sorption, solubility, hygroscopic expansion

## Abstract

When PMMA denture base acrylics are exposed to oral environments for prolonged periods, the denture base absorbs water, which has a negative influence on the denture material and the degree to which the denture base will be clinically effective. This study assessed the water sorption, desorption, and hygroscopic expansion processes within PMMA denture-base resins reinforced with nanoparticles or fibre in comparison to the non-reinforced PMMA. The surfaces of the fillers were modified using a silane coupling agent (y-MPS) before mixing with PMMA. Group C consisted of specimens of pure PMMA whereas groups Z, T, and E consisted of PMMA specimens reinforced with ZrO_2_, TiO_2_ nanoparticles, or E-glass fibre, respectively. The reinforced groups were subdivided into four subgroups according to the percentage filler added to the PMMA resin by weight (1.5%, 3.0%, 5.0%, or 7.0%). Five specimens in disc shape (25 ± 1 mm × 2.0 ± 0.2 mm) were tested for each group. To assess water sorption and hygroscopic expansion, specimens from each group were individually immersed in water at 37 ± 1 °C for 180 days. The samples were then desorbed for 28 days at 37 ± 1 °C, to measure solubility. Water sorption and solubility were calculated using an electronic balance in accordance with ISO Standard 20795-1, and hygroscopic expansion was measured using a laser micrometre. Statistical analysis was undertaken at a *p* ≤ 0.05 significance level using a one-way ANOVA followed by Tukey post-hoc tests. The results demonstrated that the values of sorption (W_sp_), mass sorption (M_s_%), and % expansion within the tested groups reached equilibrium within 180 days. A noticeable difference was observed in groups Z and E for (W_sp_)/(M_s_%) compared to the Group C, but this was not significant. However, the difference between Group C and Group T for these measurements was significant. Non-significant differences also existed between each respective reinforced group and the control group in terms of hygroscopic expansion % values. During the 28-day desorption period, there were no differences in the values of solubility (W_sl_)/mass desorption (M_d_%) between Group C and each of the reinforced tested groups. The findings indicate that the inclusion of ZrO_2_ nanoparticles or E-glass fibres does not increase the water solubility/sorption of the PMMA. However, modifying the PMMA with TiO_2_ did significantly increase the water sorption level.

## 1. Introduction

Due to its favourable properties, polymethylmethacrylate (PMMA) is commonly used to fabricate denture bases [1,2]. However, the characteristics of PMMA denture bases can be negatively influenced by aqueous environments, such as water or saliva, during its clinical use [3,4,5]. There is an inherent need to ascertain how much water denture base materials are absorbed through the surface into the material [5,6,7,8]. Polymer molecules have polar properties; as such, PMMA denture resin slowly absorbs water, depending on the homogeneity of the polymer matrix at the time of water exposure [4,5,6,7]. The absorbed water disperses through the polymer matrix and may soften the denture base [9,10,11]. This process has a plasticising effect on the PMMA denture base, thereby reducing its strength [6,7,12]. Water sorption/solubility can also cause colour [13] and dimensional instability [8,13], and negatively impact the biocompatibility of the denture base materials [14]. As water absorption is directly associated with the durability of denture bases, it also impacts their clinical success [15]. Therefore, there is a need to minimise the amount of water absorbed by a denture base material [16]. Water diffuses across the polymer matrix, potentially causing volumetric hygroscopic expansion when it occupies the inter-chain free volume and micro-voids [3,8,10,17]. This represents partial compensation for the negative influence of polymerisation shrinkage during the production of heat-polymerised denture bases, which shrinks the polymer base [6,18,19]. Volumetric changes expose the resin to internal stress, which can lead to the formation of cracks (micro and macro). These cracks may ultimately cause the denture fracture [2,9]. The extent to which water diffuses within the polymer-based material is influenced by the presence of voids in the polymer matrix, the degree of polymerisation, and operating temperature [20,21]. Solubility, the reverse of the sorption process, is responsible for material loss when a material is immersed in a liquid [7,16]. The solubility of a polymeric material depends on the level of water-leaching monomer present in the oral cavity [10,11]. Residual monomer can leach out of material into the oral fluids and subsequently cause tissue irritation [6,7,16]. There is a positive correlation between water sorption and residual monomer content [22]. When residual monomer is present, the solubility and sorption of the material may increase [9].

When fabricating polymers, researchers have typically aimed to produce denture resins that have low levels of solubility, low water sorption, and increased dimensional stability [9]. Denture base resins with these favourable characteristics have consistently maintained their appearance and performance over long timeframes. In addition, they do not leach material in a way that could potentially undermine the functional performance of the material [23]. The sorption and solubility behaviours of polymer resins can be influenced by several factors including the type of filler, filler content, polymeric matrix composition, and degree of conversion [16,24]. The water solubility and sorption of denture polymers were determined in line with the ISO 20795–1 standard [25]. The water sorption of heat-cured materials should not exceed 32 μg/mm^3^ and the loss in mass per unit volume (soluble material) should not go beyond 1.6 μg/mm^3^ [25].

Several researchers have aimed to enhance the mechanical properties of PMMA denture bases by incorporating fillers into such constructs [26,27,28,29] as opposed to enhancing the physical properties of the PMMA itself [30]. Nanoparticles have been increasingly used as fillers for polymer reinforcement due to their high flexural strength, corrosion and abrasion resistance, excellent toughness, and biocompatibility [31,32]. Studies have consistently found that glass fibres, such as electrical glass (E-glass), represent the most suitable option for dental applications because of their positive aesthetic qualities and the strong bonds they form with the polymer matrix [27,33]. Thus, incorporating fibres into resin composites has been found to be beneficial [4,34].

The physical properties of PMMA have a direct impact on the mechanical properties of the denture base and the subsequent clinical performance. However, there is a lack of studies and in-depth data available regarding the effect E-glass fibre or nanoparticle fillers have on the water sorption and solubility of heat-cured PMMA denture bases. Thus, the primary aim on this work was to evaluate the time-dependent water sorption and associated behaviours of the heat-cured PMMA denture bases that contained various concentrations of fillers and different type fillers in comparison to the pure PMMA resin. The null hypotheses were that incorporating (1) different type of fillers and (2) different concentrations of each filler into the heat-cured PMMA resin would not significantly affect sorption or solubility and hygroscopic expansion properties.

## 2. Materials and Methods

### 2.1. Materials

Heat-polymerised acrylic resin (Lucitone-199^TM^, Dentsply International, York, PA, USA) that consisted of a liquid monomer and powder polymer was supplemented with three types of filler material: Silanised E-glass fibre (3 mm in length, 15 μm in diameter, Hebei Yuniu Fiberglass, Xingtai, China), TiO_2_ nanoparticles (Titanium(IV) oxide, anatase, nanopowder, <25 nm particle size, ≥99.5% trace metals basis, Sigma Aldrich, Gillingham, UK), and ZrO_2_ nanoparticles (Zirconium(IV) oxide-3 mol % yttria stabilised, nanopowder, <100 nm particle size, Sigma Aldrich, Gillingham, UK). For the purposes of silanisation, a silane coupling agent (3-(Trimethoxysilyl) propyl methacrylate, assay 98% Sigma Aldrich, Gillingham, UK) and ethanol (Ethanol, absolute (C_2_H_6_O, EtOH) Fisher Scientific, Loughborough, UK) were utilised.

### 2.2. Methods

#### 2.2.1. Specimen Preparation

A brass mould was used that contained nine circular cavities, each measuring 25 ± 1 mm diameter × 2.0 ± 0.2 mm thickness. The mould was utilised to fabricate sixty-five disc-shaped specimens (*n* = 5/group) in total. These dimensions represented a slightly modified version of the specimen standards outlined in ISO 20795-1 [25]. Two types of nanoparticles (ZrO_2_ and TiO_2_) and one type of fibre (E-glass) were individually added to the PMMA acrylic resin at different weight concentrations (1.5 wt.%, 3.0 wt.%, 5.0 wt.%, and 7.0 wt.%). The specimens were divided into four main groups: Group C (PMMA only control group), and groups Z (ZrO_2_), T (TiO_2_), and E (E-glass fibre). The latter three groups were further divided into four subgroups according to their filler concentrations as follows: 1.5 wt.% ZrO_2_, 3.0 wt.% ZrO_2_, 5.0 wt.% ZrO_2_, 7.0 wt.% ZrO_2_; 1.5 wt.% TiO_2_, 3.0 wt.% TiO_2_, 5.0 wt.% TiO_2_, 7.0 wt.% TiO_2_; 1.5 wt.% E-glass fibre, 3.0 wt.% E-glass fibre, 5.0 wt.% E-glass fibre, and 7.0 wt.% E-glass fibre.

#### 2.2.2. Silanisation of Nano-ZrO_2_ and Nano-TiO_2_

The particle surface was impregnated with silane coupling agent (γ-MPS) to facilitate the formation of a bond between the PMMA resin matrix and the inorganic filler particles and create reactive groups on the surface of the PMMA. The treatment was performed by individually adding 15 g of ZrO_2_ and TiO_2_ nanoparticles to 70 mL of ethanol and mixing them in a plastic container at a speed of 1500 rpm (DAC 150.1 FVZK, High Wycombe, Buckinghamshire, UK). γ-MPS 0.45 g (3.0 wt.%) was subsequently introduced to the solution, which was then stirred at room temperature using a magnetic stirrer at a speed of 200 rpm for 2 h. After the stirring process, the mixture was divided equally into two parts and poured into 50 mL plastic tubes. The tubes were sealed with a lid and then placed into a centrifuge machine (Heraeus Co., Hanau, Germany) and rotated for 20 min at a speed of 4500 rpm at a temperature of 23 °C. This process separated the ethanol, resulting in sediment that contained silanised nanoparticles. The nanoparticle materials were subsequently dried in a Genevac machine (Genevac EZ-2 series, SP Scientific Company, Ipswich, UK) for 3 h at 50 °C [28].

#### 2.2.3. Blending Filler with PMMA/MMA

The heat-polymerised acrylic resin was prepared at a ratio of 21 g/10 mL in line with the manufacturer’s instructions. The silanised filler materials were weighed using a precision digital balance (Ohaus Analytical, Parsippany, NJ, USA) and added to the heat-cured acrylic according to the filler concentrations. Using a speed mixer at a speed of 1500 rpm for 10 min, MMA monomer with nanoparticles were mixed to produce a modified monomer. The modified monomer was then combined with PMMA powder. To produce the silanised E-glass fibre samples, the fibres were wetted with 4.3 mL of MMA, and 0.4 g of the PMMA powder was then mixed with MMA liquid for 10 s. To ensure the materials were thoroughly mixed, the process was repeated six times. The resultant solution underwent additional stirring for a further 2 min to make sure the fibres were adequately embedded within the MMA matrix. The remaining resin powder (9.6 g) and the remaining monomer (MMA) were added to the fibre-PMMA mixture [28].

In both cases, the PMMA was incorporated within the modified MMA (monomer + filler). A spatula was employed to ensure the PMMA powder was adequately moistened in the modified monomer. Once the PMMA powder and altered monomer had reached the “dough” stage, it was packed into the mould. The mould was then sealed and inserted into a hydraulic press (Sirio Dental, Meldola, Italy), and pressure applied up to 10.34 MPa. The mould was then place in a flask and the specimens were polymerised in a water bath based curing unit (Wassermann Dental-Maschinen GmbH, Hamburg, Germany) at room temperature. The temperature of the unit was gradually increased to 74 ± 1 °C for 1.5 h and then increased to 95 °C for 30 min. After completing the polymerisation of the specimens, the flask was set aside for 30 min to cool before being opened to extract the specimens. The specimens were then trimmed using a grinder (MetaServ 250, Buehler Ltd., Esslingen, Germany) and polished with graded SiC abrasive papers (600–, 800–, and 1000–grit) (Buehler Ltd., Esslingen, Germany) to prepare specimens with smooth surfaces [28].

#### 2.2.4. Water Sorption and Solubility Measurement

According to ISO Standard 20795-1 [25], the specimens were inserted into a glass desiccator with fresh silica gel, which, in laboratory incubators, works as a dehydrator at 37 ± 1 °C. The specimens were left in the desiccator for 24 h, then placed into another desiccator at room temperature for 60 min. The samples were then weighed with a calibrated electronic analytical balance (Ohaus Analytical, Parsippany, NJ, USA) accurate to within ±0.001 mg. The cycle of desiccation continued until the weight of the specimens varied by no more than 0.2 mg over any 24 h period, thus demonstrating that they were completely dehydrated and had reached the constant mass. The constant mass (m_1_) achieved represented the initial mass for all specimens. The volumes (V) for every specimen in cubic millimetres were calculated by measuring the mean of the diameters of the specimens at three locations and the thickness of the specimens at five different locations: one being the centre, the remaining four being evenly distributed points around the circumference using a digital caliper (Absolute Digimatic, Mitutoyo Corp, Kanagawa, Japan). Equation (1) was used to calculate the volume:

(1) $$V=\pi r^{2}h$$

where r is the circular disc’s radius in millimetres and h is the disc’s height in millimetres.

The specimens of each group (*n* = 5) were stored in individual glass containers containing 15 mL distilled water at 37 ± 1 °C for 180 days. For the first week, these specimens underwent daily weighing and were then weighed at 14, 21, 28, 60, 90, 120, 150 and then 180 days, the point at which water sorption equilibria was achieved. At each time point, the specimens were carefully taken from the water employing tweezers, dried with hand towels until no visible moisture was present, air dried for 15 s and then weighed a minute later before being returned to their containers. In order to maintain constant pH of the water, fresh distilled water was poured into the containers after every measurement [35]. The mass as recorded was noted as m_2_ (*t*) (*t* = time).

Once the 180-day water sorption weighing process had been completed, the specimens from each group underwent reconditioning in a desiccator as per the process previously described for the water sorption measurement. A cycle was taken after Day 1, 7, 14, 21, and 28, and the mass at these testing points was noted as m_3_ (*t*). A constant mass was reached at Day 28. Equations (2) and (3) were used to calculate the water sorption (W_sp_) and the solubility (W_sl_) in μg/mm^3^ for each specimen in different groups.

(2) $$w_{\mathrm{sp}}=\frac{m_{2\;\left(t\right)}-m_{3}}{v}$$

(3) $$w_{\mathrm{sl}}=\frac{m_{1}-m_{3\;\left(t\right)}}{v}$$

where m_1_ represents the conditioned mass of the specimen in micrograms (μg), m_2_ is the specimen mass post-water immersion in micrograms (μg), m_3_ is the recondition specimen’s mass (μg) and v is the volume of specimen (cubic millimetres).

Equations (4) and (5) were used to calculate the percentage mass change (M_s_%) and percentage solubility (M_d_%) as representative of the total mass loss of components, respectively [10].

(4) $$\mathrm{Sorption}\;\mathrm{mass}\;\mathrm{change}\;\left(M_{s}\%\right)=\frac{m_{2\;\left(t\right)}-m_{1}}{m_{1}}\times 100$$

(5) $$\mathrm{Desorption}\;\mathrm{mass}\;\mathrm{change}\;\left(M_{d}\%\right)=\frac{m_{3\;\left(t\right)}-m_{1}}{m_{1}}\times 100$$

#### 2.2.5. Measurement of Hygroscopic Dimensional Changes

A custom-made non-contact laser micrometre was employed to measure the hygroscopic expansion of the specimens. This instrument utilised a laser-scan micrometre (LSM) system, with a heavy stainless steel base mount. The sample holder in the instrument can be rotated stepwise around a horizontal plane using an electronic stepper-control unit. During stepwise rotation, the LSM took measurements of the sample diameters. The diametral value for each specimen at each measuring point represents an average of 300 data values [10]. Each specimen’s initial mean diameter was assessed and noted down as d_1_. The mean diameter assessed at each point (*t*) of water sorption was noted as d_2(*t*)_. Prior to specimen diameter measuring, care was exercised in removing each specimen, drying it on a filter paper for one minute, and then placing it on the specimen holder. Once the change in diameter had been measured, the specimens were returned to water [10]. Equation (6) was used to determine the percentage change in the diameter of the specimen following immersion in water.

(6) $$d\left(\%\right)=\frac{d_{2}{}_{\left(t\right)}-d_{1}}{d_{1}}\times 100$$

Equation (7) was used to determine the volumetric change (V(%)) in the specimens in accordance with the assumption of isotropic expansion behaviour.

(7) $$V\;\left(\%\right)=\left[{\left(1+\frac{d\left(\%\right)}{d_{1}}\right)}^{3}-1\right]\times 100$$

where d_1_ represents the mean diameter prior to water immersion and d_2_(*t*) represents the mean diameter during water immersion as recorded at set time intervals.

### 2.3. Statistical Analysis

The statistical software (*SPSS statistics*, Version 25, IBM Corp. 2017, New York, NY, USA) was used to perform statistical analysis. According to the outcomes of the Shapiro–Wilk and Levene tests, the p-values were not statistically significant at the *p* > 0.05 level, thereby suggesting that the data exhibited homogeneity and was normally distributed. One-way ANOVA was performed at a *p* ≤ 0.05 significance level to ascertain the significance of any subgroup variations compared to the control specimens in terms of water sorption, hygroscopic expansion after 180 days, and mass change. A Tukey post-hoc test was then conducted to identify any significant differences that could be observed within a given group. The same statistical analysis was performed to assess the solubility and mass loss differences between the samples by evaluating weight changes after the 28-day desorption cycle.

## 3. Results

**Figure 1:**
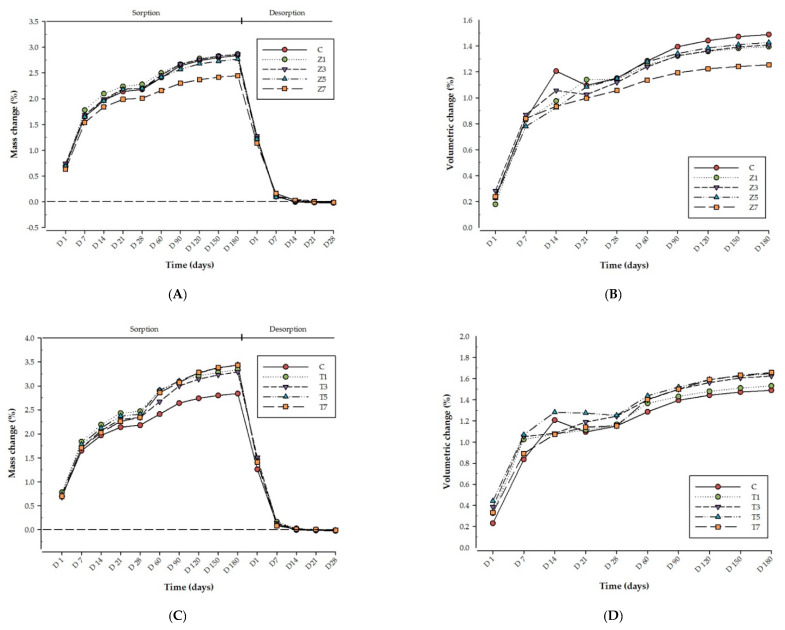

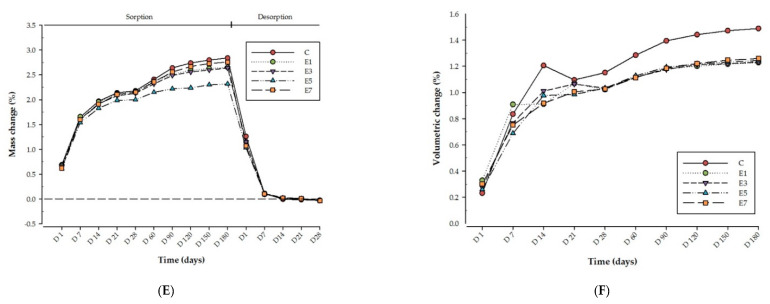
Line graph illustrating the mass change and hygroscopic expansion of each respective tested group with control group (**A**) mass change for Z group, (**B**) volumetric change for Z group, (**C**) mass change for T group, (**D**) volumetric change for T group, (**E**) mass change for E group, (**F**) volumetric change for E group.

The means and standard deviations of sorption and solubility values are presented in Table 1 and Figure 1. The largest amount of mass and dimensional changes was observed between Day 1 and 7 in all groups. The mass and dimensional magnitudes of water uptake in all groups subsequently increased gradually until they reached equilibrium on Day 180. The mass of the groups rapidly reduced during the desorption cycle until a constant mass was achieved by Day 28. Data on Day 180 of immersion in distilled water indicated that there were no significant variations in the sorption mass change percentage (M_s_%) or water sorption (W_sp_) in the Z and E groups in comparison to the control Group C (*p* > 0.05). However, M_s_% and W_sp_ of the T groups were significantly higher than those observed in the control group (*p* < 0.05). Data after 28 days of desorption revealed that there were no significant differences in the percentage desorption mass change (M_d_%) or water solubility (W_sl_) between the control group and each respective reinforced tested group (*p* > 0.05). Furthermore, no significant variations in the volumetric shrinkage values (V(%)) between the control group and each respective reinforced tested group were found (*p* > 0.05).

The water sorption within the Z groups ranged from 27.9 to 32.0 µg/mm^3^ at Day 180 (Table 1). The greatest sorption was observed in Z%3 (32.0 µg/mm^3^) followed by Z%1.5 (31.1 µg/mm^3^), Z%5 (30.9 µg/mm^3^), and Z%7 (27.9 µg/mm^3^). No significant differences were observed between the specimens in the Z subgroups themselves or between the Z groups and Group C (31.6 µg/mm^3^; *p* ≥ 0.05). The mass according to water uptake of all the specimens within Group C and the Z groups increased by various degrees until a point of equilibrium was reached at Day 180. As the concentrations of ZrO_2_ nanoparticles increased, at Day 180, the percentage mass changes in the ZrO_2_-reinforced specimens gradually fell by 2.87, 2.84, 2.73 to 2.44%, respectively (Figure 1A). No significant variations (*p* > 0.05) were observed between the specimens within the ZrO_2_-reinforced groups. The dimensional change percentages of the specimens within the ZrO_2_-reinforced groups marginally reduced 7.38%, 5.37%, 4.03%, and 16.11% at Z%1.5, Z%3, Z%5, and Z%7, respectively by comparison to the control group (1.49; *p* = 0.626). However, no significant variations (*p* > 0.05) were observed within these groups. The solubility measurements of the specimens within the Z groups varied from 0.13 to 0.15 µg/mm^3^. As can be seen in Table 1, these values were not significantly lower than those observed within Group C (0.27 µg/mm^3^; *p* = 0.65). The percentage mass according to water loss (M_d_%) of all the specimens in the Z groups swiftly reduced until a constant mass was achieved at Day 28. Again, no significant differences in M_d_% were observed across these groups.

By contrast, W_sp_ and M_s_% were significantly higher in the T groups when compared to Group C (31.6 µg/mm^3^ and 2.84%, respectively; *p* < 0.05) at Day 180, as presented in Table 1 and Figure 1B. However, no significant variations (*p* > 0.05) in W_sp_ and M_s_% were found within the T groups. As the ratio of TiO_2_ nanoparticles increased by 1.5 wt.%, 3.0 wt.%, 5.0 wt.%, and 7.0 wt.%, the water sorption of TiO_2_-reinforced groups gradually increased to 35.6, 35.9, 37.8, and 38.5µg/mm^3^, respectively. At Day 180, the percentage mass due to water uptake of the specimens within the TiO_2_-reinforced groups ranged from 3.33% to 3.44%. The dimensional change percentages within the T groups increased non-significantly by 2.68%, 8.72%, 10.73%, and 11.41% at T%1.5, T%3, T%5, and T%7 concentrations, respectively, in comparison to Group C (1.49; *p* > 0.05). Furthermore, no significant variations (*p* > 0.05) were noted within the T groups. The solubility values (W_sl_) of the T groups gradually decreased when the TiO_2_ nanoparticles concentration increased, as represented in Table 1. However, no significant variations in W_sl_ were noted between the T groups and Group C, or between the T groups themselves. The percentage mass of all the T groups reduced until a constant mass was reached at Day 28. However, no significant differences in M_d_% were found within these groups.

At Day 180, no significant variations in water sorption (W_sp_) were evident between the samples within the E groups or between the E groups and Group C (31.6 µg/mm^3^; *p* ≥ 0.05) (Table 1). The highest sorption level was seen in E%7 (31.9 µg/mm^3^), while the lowest was observed in E%5 (27.7 µg/mm^3^). At Day 180, all specimens within the E groups exhibited a non-significant lower percentage mass increase in comparison to those in Group C (2.84%; *p* ≥ 0.05). The percentages of dimensional changes of the specimens within the E-glass fibre-reinforced groups exhibited a marginal reduction of 14.76%, 17.45%, 16.78%, and 15.44% at E%1.5, E%3, E%5, and E%7, respectively, when compared to the control group (1.49; *p* = 0.626). No significant differences (*p* > 0.05) in dimensional change were noted in the samples within the E groups. The solubility values of the specimens (W_sl_) in the E groups steadily increased in correlation with an increase in the concentration of E-glass fibre as follows: E%1.5 = 0.26 µg/mm^3^, E%3 = 0.30 µg/mm^3^, E%5 = 0.33 µg/mm^3^, and E%7 = 0.43 µg/mm^3^ (Table 1). However, no significant differences in W_sl_ were recorded between the specimens in the E-glass fibre-reinforced groups and those in Group C, or between the specimens within the E-glass fibre-reinforced groups. The percentage decrease of mass change in the E-glass fibre-reinforced groups dropped until a constant mass was evident on Day 28. No significant variations in M_d_% were noted within these groups.

## 4. Discussion

Although enhancing the mechanical characteristics of acrylic resins used for manufacturing denture bases [28] is important for clinical purposes, the material’s solubility and water sorption properties must not be compromised. The structure and function of polymeric materials can be affected by sorption and solubilisation of water [36]. Dentures made from acrylic tend to absorb water over time due to the molecular polarity of the polymer within the material [6,37]. As a result of diffusion, water enters the material and interferes with the bonds between the polymer chains (hydrolytic degradation of polymers) [4,37,38]. This can stimulate dimensional changes as a result of resin swelling, a reduction in mechanical properties due to softening, and unreacted monomer leakage. All these factors can reduce the service life of the bases [6,36,39]. Thus, there is a requirement to investigate hygroscopic and hydrolytic effects in more depth.

In our previous studies, the effects of incorporating ZrO_2_ and TiO_2_ nanoparticles or E-glass fibre at various concentrations (1.5, 3.0, 5.0 and 7.0 wt.%) on the flexural strength, hardness, fracture toughness, and impact strength of PMMA denture base were examined [28,29]. The findings revealed that the optimal filler concentrations for significantly increasing the flexural strength of PMMA denture base were 3.0 wt.% ZrO_2_, and 5.0 wt.% and 7.0 wt.% E-glass fibre. The optimal filler concentration for significantly increasing the surface hardness of the PMMA was 3.0 wt.% and higher [28]. Furthermore, 1.5 and 3.0 wt.% ZrO_2_ and 1.5 wt.% TiO_2_, and all E-glass fibre concentrations significantly increased the fracture toughness of the PMMA. Incorporating ZrO_2_ or TiO_2_ nanoparticles at any concentration did not enhance the impact strength of the PMMA, but adding 3.0, 5.0, and 7.0 wt.% of E-glass fibres did significantly increased the impact strength [29].

In this study, after six months of storing test samples in distilled water, PMMA heat-cured modified with three different fillers; ZrO_2_ nanoparticles, TiO_2_ nanoparticles or E-glass fibre were evaluated for water sorption, solubility, and hygroscopic expansion characteristics. The outcomes indicated no significant variations between each respective reinforced group (ZrO_2_ nanoparticles/E-glass fibres) and the control group in terms of any of the properties tested with the exception of M_s_% and W_sp_ of the TiO_2_ nanoparticle-reinforced groups, which were significantly higher than those observed in the control group. Therefore, the first hypothesis was rejected. However, the second hypothesis was accepted on the basis that there were no significant variations within each of respective reinforced groups (Z, T or E).

Although studies have been performed on solubility and water sorption [7,9], the results of prior investigations cannot be reliably compared because the study periods, storage medium, sample dimensions, and equilibrium periods of water sorption are different [40]. In most studies, either water or artificial saliva were used as the storage medium [16,35]. However, testing indicates that the use of water as opposed to artificial saliva, and vice versa, does not have a significant impact on the values of the tested properties [16]. In this experiment, distilled water was used since it had a similar degrading effect to artificial saliva. Artificial saliva and distilled water, however, do not accurately reflect oral environmental conditions [41]. In this study, water was replaced regularly between measurements to prevent pH fluctuations, which may have affected sorption and solubility [35]. Figure 1 indicates that the amount of water gain progressively increased for all tested materials during the first 60 days. However, all the tested materials gained the most water during the first week because most of the residual monomer within the samples leached from the PMMA during the first few days of storage [7]. This finding was consistent with many prior studies from the perspective of maximum amount of water sorption observed during the first seven days [16,17].

The findings of previous research revealed that the incorporation of fibres and nanoparticles in PMMA to improve its mechanical properties [28,42], could increase the water sorption of PMMA acrylic resins [43,44]. The degree of water sorption exhibited by a resin can vary according to a range of factors, including filler type (particles/fibres), polymer (monomer, degree of conversion), dispersion of fillers in the matrix, and filler size [24,40]. According to Miettinen and Vallittu [45], the solubility and water sorption of polymers varied with material homogeneity. The higher the homogeneity of the material, the less soluble it is and the less water it absorbs. Additionally, water can potentially penetrate the material via gaps in the filler surface and the bonds between the filler and the resin matrix [6,36]. A silane-coupling agent can be employed to increase the interfacial interaction between the resin matrix and the filler particles, thereby improving its mechanical and physical properties [39,46]. Furthermore, there is also a requirement to ensure the fillers are evenly dispersed throughout the polymer matrix [16]. In the current study, a strong bond was created between the filler and resin matrix by treating the fillers with a silane coupling agent. Two additional factors that can significantly impact the solubility and water sorption of a material are hydrophilicity and crosslinking of the polymer network structure [17,36,47]. Moreover, the level of solvent the resin-composite absorbs during the exposure period varies according to the nature of the filler matrix and the porosity of the material [36,47].

As can be observed in Table 1, the water sorption in the specimens in the ZrO_2_-reinforced groups was between 27.9 and 32.0 µg/mm^3^. These values were a little lower than those reported in Group C. However, the variations were not statistically significant. These findings are aligned with those of Saleh et al. [16], who examined the water sorption and solubility of high-impact heat-cured PMMA reinforced by different concentrations of ZrO_2_ nanoparticles after immersion in distilled water and artificial saliva over six months. They concluded that there were no significant differences between the reinforced groups and the control group, except for specimen with 10 wt.% ZrO_2_ in artificial saliva, which exhibited a significantly higher solubility [16]. However, Asar et al. [6] found that the incorporation of ZrO_2_ or TiO_2_ significantly reduced the water sorption and solubility of the resins when compared to non-reinforced PMMA acrylic resins. The percentage mass change in the ZrO_2_-reinforced specimens decreased slowly as the concentration of ZrO_2_ nanoparticles increased (Figure 1A). Ergun et al. [43] determined that higher ZrO_2_ concentration led to higher water absorption of the PMMA/ZrO_2_ composite. The solubility of the specimens in the ZrO_2_-reinforced groups was between 0.13 and 0.15 µg/mm^3^, which was lower than those found in Group C (Table 1). However, again the differences were not statistically significant. The relatively low enhancement in solubility might be attributed to the incorporated ZrO_2_ nanoparticles that fill any potential spaces within the PMMA resin [16] and thereby decreasing the water sorption and mass change within the samples. Our previous SEM images displayed the fracture surfaces of 7.0 wt.% ZrO_2_ nanoparticle specimens and revealed that this material was less brittle compared to the control group specimens [28,29].

The results in terms of the water sorption and solubility of the E-glass fibre-reinforced groups were comparable to those of the ZrO_2_-reinforced groups, with no significant variations in water sorption compared to the Group C. However, Table 1 presented that the E%1.5, E%3, and E%5 groups exhibited lower water sorption values than the respective specimens in the Group C. Furthermore, the decrease in percentage mass of the E-glass fibre-reinforced groups was not significantly lower than the control group. There was an observable correlation between an increase in the concentration of the E-glass fibre and the solubility values observed within the specimens in Group C (Table 1). However, these values were not deemed to be statistically significant. Polat et al. [4] observed that reinforcing denture base PMMA resin with short glass fibres reduced the water solubility and sorption. According to Miettinen and Vallittu [7], heat-cured PMMA reinforced with glass fibre exhibited lower water absorption values than the non-reinforced PMMA. Both these findings align with the results of the current study. The relatively low values could be attributed to the lack of voids in the structure of the resin composite, which prevents water ingress into the richly impregnated areas of the test specimens [2,7]. An additional explanation could be that the use of a silane coupling agent promoted better bonds between the glass fibre/ZrO_2_ nanoparticles and the polymer matrix [6,38]. This was supported by the SEM images in the previous work, which confirmed that little or no gap had formed around the fibres on the fracture surface of the E-glass fibres specimens [28,29].

This study found that increased TiO_2_ content significantly increased water sorption and the percentage mass of the TiO_2_-reinforced groups when compared with the non-reinforced PMMA. However, as the percentage of TiO_2_ nanoparticles increased, the solubility values for TiO_2_-reinforced groups decreased slightly. The decrease was not significant compared to Group C. Several factors might account for the higher water sorption values, including TiO_2_ particle size, non-homogeneous distribution, and the weak bond between the nano-TiO_2_ filler and PMMA resin matrix, which enabled water to penetrate the matrix [43,44]. A polymer chain can develop porosity when the acrylic resin is polymerised [6]. The porosity of a polymer, as well as its micro-voids, act as the sites for molecule sequestration and enhance solvent uptake, allowing fluid to flow into and out of the polymer [36]. As a result, the higher values of water sorption in these specimens could also be attributed to the presence of an increased number/size of voids. High solubility is not always associated with high absorption of water [35]. In most cases, the resin’s water solubility is a result of the leaching of free residual monomers, additives, fillers, and filler components [35]. There is a correlation between the degree of conversion and the amount of leachable the unreacted monomers; the higher the degree of conversion, the lower the quantity of unreacted monomers, and thus the lower the solubility [35,48]. Water forming clusters in an oversaturated monomer system deteriorates the mechanical properties of the material because they behave like microvoids [38]. These findings were in agreement with the SEM images in our previous studies, which revealed that the fractured surface of TiO_2_ nanoparticles exhibited indications of particle agglomeration with small voids at 7 wt.% that might lead to the formation of a poor bond between PMMA matrix and TiO_2_ nanoparticles [28,29]. As a result, incorporating TiO_2_ nanoparticles into PMMA reduced its flexural strength [28]. However, this study examined how the incorporation of TiO_2_ filler into PMMA denture base materials could impact the physical properties of the material [28]. The increase in mass could be the result of residual monomer being released during storage over a long period of time. At each measurement, this monomer was replaced with the distilled water, leading to enhanced sorption, especially in the control group specimens. However, the incorporating of ZrO_2_ nanoparticles and E-glass fibre in the resin could decrease the level of residual monomer in the filler-composite resin, as indicated by observation that the specimens in these groups exhibited lower sorption than the control group [16].

During immersion in water, the dimensions of the materials change in relation to water uptake [48]. Water absorption causes the resin to expand (swell), thereby forcing the macromolecules apart [4,36]. Chow et al. [49] studied polyethylene fibre-modified PMMA denture resin when immersed in water, and they observed a significant reduction in water sorption and dimensional changes. In addition, Wong et al. [48] assessed the dimensional behaviour of dentures in response to water sorption and release. According to their results, the water sorption and solubility of the denture bases resins could affect the dimensions of the denture base resins [48]. Ladizesky et al. [50] also examined the water sorption and dimensional changes observed in woven polyethylene fibre-reinforced denture base acrylic resins. They stated that the inclusion of a higher fibre content insignificantly decreased water sorption. Therefore, the incorporation of fibre in resins does not notably impact the water sorption of heat-cured acrylic [50]. The results of the current study indicated that the inclusion of fibres or nanoparticles in PMMA did not significantly alter the dimensional accuracy of the specimens. A non-significant reduction in the percentages of dimensional changes of the specimens within the ZrO_2_-and E-glass fibre-reinforced groups was observed when compared to the control group. However, in the TiO_2_-reinforced group, the dimensional change percentage did increase non-significantly when compared to the Group C values. Dimensional changes could be influenced by several factors, such as the thickness and size of the dentures [4]. Acrylic resin, filler, filler content, and the processing method directly influence dimensional change.

### Clinical Significance

The outcomes of the current study indicate that the incorporating the tested nanoparticles or E-glass fibre within PMMA denture base resins does not significantly reduce the amount of water absorbed by the specimen and its solubility.

## 5. Conclusions

This study focused on assessing the overall degradation resistance of three types of filler-reinforced PMMA at various filler concentrations (1.5 wt.%, 3.0 wt.%, 5.0 wt.%, or 7.0 wt.%) when stored in water for six months. Within the limitations of this study, it can be concluded that adding ZrO_2_ nanoparticles or E-glass fibres to PMMA does not have a significant positive effect on water sorption and solubility based on the filler concentrations employed in this study. However, the incorporation of TiO_2_ nanoparticles causes unfavourable changes in the PMMA denture base in terms of water sorption property.

## Figures and Tables

**Table 1:** Water sorption (W_sp_), sorption mass change (M_s_%) and hygroscopic expansion (%) after immersion period of 180 days in distilled water; Water solubility (W_sl_) and desorption mass change (M_d_%) after 28 days desorption for all tested groups presented as means and standard deviations.

Materials Group		W_sp_ (µg/mm^3^)	M_s_%	W_sl_ (µg/mm^3^)	M_d_%	Hygroscopic Expansion (%)
Control	C	31.6 (4.9) ^ac^	2.84 (0.48) ^ac^	0.27 (0.17) ^abc^	−0.024 (0.016) ^abc^	1.49 (0.18) ^abc^
ZrO_2_	Z%1.5	31.1 (6.8) ^a^	2.87 (0.68) ^a^	0.15 (0.13) ^a^	−0.014 (0.012) ^a^	1.38 (0.29) ^a^
	Z%3	32.0 (5.3) ^a^	2.84 (0.51) ^a^	0.13 (0.48) ^a^	−0.011 (0.004) ^a^	1.41 (0.20) ^a^
	Z%5	30.9 (5.8) ^a^	2.73 (0.55) ^a^	0.14 (0.17) ^a^	−0.013 (0.015) ^a^	1.43 (0.26) ^a^
	Z%7	27.9 (3.6) ^a^	2.44 (0.32) ^a^	0.13 (0.07) ^a^	−0.012 (0.006) ^a^	1.25 (0.16) ^a^
TiO_2_	T%1.5	35.6 (0.7) ^b^	3.33 (0.08) ^b^	0.22 (0.20) ^b^	−0.021 (0.020) ^b^	1.53 (0.33) ^b^
	T%3	35.9 (0.7) ^b^	3.29 (0.09) ^b^	0.16 (0.17) ^b^	−0.015 (0.017) ^b^	1.62 (0.37) ^b^
	T%5	37.8 (0.5) ^b^	3.43 (0.08) ^b^	0.12 (0.15) ^b^	−0.011 (0.014) ^b^	1.65 (0.25) ^b^
	T%7	38.5 (0.7) ^b^	3.44 (0.07) ^b^	0.11 (0.06) ^b^	−0.010 (0.005) ^b^	1.66 (0.41) ^b^
E-glass fibre	E%1.5	29.6 (6.2) ^c^	2.65 (0.64) ^c^	0.26 (0.32) ^c^	−0.023 (0.027) ^c^	1.22 (0.11) ^c^
	E%3	30.0 (6.4) ^c^	2.64 (0.68) ^c^	0.30 (0.06) ^c^	−0.026 (0.005) ^c^	1.23 (0.08) ^c^
	E%5	27.7 (3.3) ^c^	2.32 (0.26) ^c^	0.33 (0.13) ^c^	−0.028 (0.011) ^c^	1.24 (0.12) ^c^
	E%7	31.9 (5.0) ^c^	2.76 (0.49) ^c^	0.43 (0.11) ^c^	−0.037 (0.009) ^c^	1.26 (0.11) ^c^

Note: Same superscript letter within column represents non-significant difference between each filler reinforced group and control group (*p* > 0.05).

## Data Availability

Not applicable.
